# Active glucose transport varies by small intestinal region and oestrous cycle stage in mice

**DOI:** 10.1113/EP091040

**Published:** 2023-04-06

**Authors:** T. Sebastian Overduin, Hannah R. Wardill, Richard L. Young, Amanda J. Page, Kathryn L. Gatford

**Affiliations:** ^1^ School of Biomedicine University of Adelaide Adelaide South Australia Australia; ^2^ Robinson Research Institute University of Adelaide Adelaide South Australia Australia; ^3^ Lifelong Health Theme South Australian Health and Medical Research Institute Adelaide South Australia Australia; ^4^ Precision Medicine Theme South Australian Health and Medical Research Institute Adelaide South Australia Australia; ^5^ Adelaide Medical School University of Adelaide Adelaide South Australia Australia

**Keywords:** oestrous cycle, glucose, mouse, SGLT1, small intestine

## Abstract

Food intake changes across the ovarian cycle in rodents and humans, with a nadir during the pre‐ovulatory phase and a peak during the luteal phase. However, it is unknown whether the rate of intestinal glucose absorption also changes. We therefore mounted small intestinal sections from C57BL/6 female mice (8–9 weeks old) in Ussing chambers and measured active *ex vivo* glucose transport via the change in short‐circuit current (∆*I*_sc_) induced by glucose. Tissue viability was confirmed by a positive ∆*I*_sc_ response to 100 µM carbachol following each experiment. Active glucose transport, assessed after addition of 5, 10, 25 or 45 mM d‐glucose to the mucosal chamber, was highest at 45 mM glucose in the distal jejunum compared to duodenum and ileum (*P* < 0.01). Incubation with the sodium–glucose cotransporter 1 (SGLT1) inhibitor phlorizin reduced active glucose transport in a dose‐dependent manner in all regions (*P* < 0.01). Active glucose uptake induced by addition of 45 mM glucose to the mucosal chamber in the absence or presence of phlorizin was assessed in jejunum at each oestrous cycle stage (*n* = 9–10 mice per stage). Overall, active glucose uptake was lower at oestrus compared to pro‐oestrus (*P* = 0.025). This study establishes an *ex vivo* method to measure region‐specific glucose transport in the mouse small intestine. Our results provide the first direct evidence that SGLT1‐mediated glucose transport in the jejunum changes across the ovarian cycle. The mechanisms underlying these adaptations in nutrient absorption remain to be elucidated.

## Abstract (highlights)

- **What is the central question of this study?**
  Body mass and food intake change during the female ovarian cycle: does glucose transport by the small intestine also vary?
- **What is the main finding and its importance?**
  We have optimised Ussing chamber methodology to measure region‐specific active glucose transport in the small intestine of adult C57BL/6 mice. Our study provides the first evidence that jejunal active glucose transport changes during the oestrous cycle in mice, and is higher at pro‐oestrus than oestrus. These results demonstrate adaptation in active glucose uptake, concurrent with previously reported changes in food intake.

## 1 INTRODUCTION

Digestion and absorption of nutrients occurs within the gastrointestinal tract, where complex organic molecules are progressively digested into smaller and simpler entities as they pass through the tract. Nutrient absorption begins in the duodenum and peaks in the jejunum (Volk & Lacy, 2017). The terminal ileum acts to brake intestinal motility in the presence of high nutrient concentrations to maximise nutrient uptake in the small intestine (Karasov & Douglas, 2013; Kiela & Ghishan, 2016). The small intestine absorbs nutrients by passive and active transport, the latter via specific nutrient transporters (Kiela & Ghishan, 2016; Volk & Lacy, 2017). For example, active absorption of glucose is facilitated by sodium–glucose cotransporter 1 (SGLT1), a high affinity, low‐capacity transmembrane protein located at the brush border membrane of absorptive enterocytes along the upper third of small intestinal villi (Kiela & Ghishan, 2016). SGLT1 accounts for ∼90% of small intestinal glucose absorption under normal circumstances (Poulsen et al., 2015).

Food intake, appetite and the rate of gastric emptying change across the ovarian cycle in humans (Brennan et al., 2009; Gong et al., 1989; Lissner et al., 1988), rhesus monkeys (Kemnitz et al., 1984) and rodents (Bailey & Matty, 1972; Drewett, 1974; Petersen, 1976). Indeed, energy intake is decreased by 10–20% in humans (Brennan et al., 2009; Gong et al., 1989; Lissner et al., 1988) and food intake is halved in rhesus monkeys in the follicular compared to the luteal phase of the menstrual cycle (Kemnitz et al., 1984). Feeding behaviours also change during the oestrous cycle in mice, with a nadir in the number of feeding bouts (10% lower) and amount of time spent eating per bout (30% lower) at pro‐oestrus compared to metoestrus and dioestrus (Petersen, 1976). These food intake behaviours are likely to be mediated, at least in part, by cyclic changes in circulating oestrogen and progesterone concentrations. Whether nutrient absorption also changes during the ovarian cycle is less clear. Post‐prandial blood glucose concentrations following a standardised meal are 15% lower during the follicular compared to the luteal phase in women (Brennan et al., 2009), reflecting the combined effect of intestinal glucose uptake, hepatic first‐pass extraction, insulin and glucagon (Dimitriadis et al., 2021). As glucose absorption is a major contributor to the increase in post‐prandial glucose (Dimitriadis et al., 2021), higher post‐prandial glucose concentrations during the luteal phase support the hypothesis that intestinal glucose absorption is more rapid during this period than the follicular phase. Nevertheless, there is no direct evidence for this, reflecting the difficulties in interrogating this mechanism. We therefore optimised Ussing chamber methodology to directly measure active glucose transport across the mouse small intestine. We then used this method to characterise oestrous cycle differences in total and SGLT1‐mediated active glucose uptake in the mouse jejunum, the region of highest active glucose uptake.

## 2 METHODS

### 2.1 Ethics approval

All experiments conformed to the principles set out by Grundy (2015) and the ARRIVE 2.0 guidelines (Percie du Sert et al., 2020) and were conducted in accordance with the Australian Code of Practice for the Care and Use of Animals for Scientific Purposes (National Health & Medical Research Council, 2013). All experiments were approved by the South Australian Health and Medical Research Institute Animal Ethics Committee (SAHMRI AEC approval: SAM‐21‐049). The initial number of mice (*n* = 14) per condition provided 80% power at α = 0.05 to detect a 20% difference in short‐circuit current responses to glucose, based on variation in pilot experiments. As outcome variation decreased with protocol optimisation, fewer mice were required in later experiments, as indicated below.

### 2.2 Mice

Adult C57BL/6JSAH female mice (8–10 weeks old) were sourced from SAHMRI Bioresources and housed in home cages in groups of two to five under a 12‐h light cycle (07.00–19.00 h) and constant temperature of 23°C with ad libitum access to water and a standard laboratory chow (Teklad standard diet, Envigo, Huntingdon, UK). For dose–response and inhibitor (method optimisation) studies, mice remained group‐housed under breeding colony conditions until use. For oestrous cycle studies, mice were pair‐housed in individually ventilated cages with crinkle‐nest and cotton nestlet bedding material and cardboard tubes as enrichment. After at least a week's acclimatisation, vaginal smears were collected and assessed daily for at least a further week to determine oestrous cycle stage.

### 2.3 Tissue preparation and Ussing chamber conditions

Mice were humanely killed via cervical dislocation, a laparotomy performed and viscera exposed to ice‐cold Krebs–Ringers bicarbonate (KRB) buffer (115 mM NaCl, 2.4 mM K_2_HPO_4_, 0.4 mM KH_2_PO_4_, 25 µM NaHCO_3_, 1.2 mM MgCl_2_·6H_2_O, 1.2 mM CaCl_2_·2H_2_O) containing 5 mM d‐glucose and gassed with carbogen (95% O_2_–5% CO_2_), as described previously (Wardill et al., 2016). Peak food consumption occurs early in the dark period in mice, with a secondary peak shortly before lights on (Zhong et al., 2019). To minimise potential circadian and food‐induced variation in nutrient absorption, all mice had ad libitum access to food throughout the studies and were humanely killed for tissue collection ∼1–4 h after lights on. This timing avoided the period of rapid upregulation of SGLT1 expression at the onset of the dark period or before feeding (Iwashina et al., 2011). The small intestine was then excised, laid flat and measured unstretched, then divided into duodenum, jejunum and ileum. The duodenal–jejunal transition zone was defined by the ligament of Treitz, while the jejunal–ileal transition zone was identified by narrowing of the intestinal lumen, a reduction in smooth muscle thickness, a change in tissue translucency reflective of thinner smooth muscle and the presence of Peyer's patches (Volk & Lacy, 2017).

The duodenum, jejunum or ileum from each mouse was gently flushed with ice‐cold KRB buffer to remove ingesta. Eight evenly spaced sections, ∼1 cm in length, were opened longitudinally and collected across the duodenum, jejunum or ileum; four sections from the proximal and four sections from the distal half of the segment were mounted for each mouse. Each section was placed between two sliders with an exposed aperture of 0.1 cm^2^ (P2303A, Physiologic Instruments, Reno, NV, USA) and mounted into Ussing chambers. In all experiments, intestinal tissues were mounted in the first chamber between 8 and 19 min after the mouse was humanely killed (usually by 15 min) and tissue mounting was completed between 17 and 29 min. All water‐jacketed chambers were maintained at 37°C, and tissues bathed in 5 ml KRB were continuously gassed with carbogen. Tissues were voltage‐clamped to zero potential difference which was maintained throughout each experiment. Short circuit current (*I_s_*_c_) was monitored and recorded continuously (Acquire and Analyse 2.3, v2.3.4, Physiologic Instruments). All tissues were equilibrated for 20 minutes prior to d‐glucose challenge. d‐Mannitol (25 mM) was added to the serosal chamber concurrent with d‐glucose addition to the mucosal chamber to provide an osmotic balance. The absolute difference in short circuit current, expressed as ∆*I*_sc_ (µA/cm^2^), was calculated as the difference between the maximum *I*_sc_ reached during the 4 min following d‐glucose challenge and the average *I*_sc_ during the 2 min prior to the d‐glucose challenge. Carbachol (100 µM), a chloride secretagogue, was added to both chambers 10 min after the d‐glucose challenge to evoke a positive *I*_sc_ flux as a marker of tissue viability (Wardill et al., 2016).

Data from individual samples were excluded if any of the following occurred: (1) a negative *I*_sc_ reading immediately prior to d‐glucose challenge, (2) high baseline drift, defined as change in *I*_sc_ of ±20 µA/cm^2^ in the 2 min preceding the d‐glucose challenge, (3) high baseline noise, defined as an average *I*_sc_ point‐to‐point variation of ≥3 µA/cm^2^ in the 2 min interval preceding the d‐glucose challenge, and (4) poor tissue viability, defined as ∆*I*_sc_ of ≤20 µA/cm^2^ in response to 100 µM carbachol.

### 2.4 Study 1: region‐specific glucose dose response

Following equilibration, tissue segments from the proximal (four segments) and distal (four segments) half of each small intestinal region were randomised and challenged with d‐glucose in the mucosal chamber. A stimulus of 5, 10, 25 or 45 mM d‐glucose was added to the basal glucose concentration of 5 mM, resulting in final d‐glucose concentrations of 10, 15, 30 or 50 mM (*n* = 5–10 observations per concentration in each region, samples from a total of 50 mice). These d‐glucose concentrations were selected based on SGLT1 saturation at ∼50 mM d‐glucose in rat jejunum (Kellett & Helliwell, 2000), and our findings of short‐circuit current reversal at higher d‐glucose concentrations (100 and 250 mM; data not shown).

### 2.5 Study 2: SGLT1 inhibition

Dimethyl sulfoxide (DMSO) was utilised as a vehicle for the SGLT1 antagonist phlorizin, and therefore we first assessed whether DMSO affected short‐circuit current responses to d‐glucose and carbachol. Small intestinal regions (duodenum, jejunum, ileum) from a total of 25 mice were prepared as described previously and incubated for 20 min in 0, 0.5 or 1% v/v DMSO in KRB buffer in the mucosal chamber, followed by a 45 mM d‐glucose challenge.

We next tested a range of phlorizin concentrations (0.1–1 mM) in jejunum to determine an appropriate concentration to attenuate SGLT1‐mediated intestinal d‐glucose transport in mice. The highest concentration tested (1 mM) was based on inhibition of glucose‐evoked release of glucagon‐like peptide‐1 (≥90%) in human ileum (Sun et al., 2017). Jejunal segments from a total of 13 mice were incubated for 20 min with DMSO (1% v/v ) containing 0, 0.1, 0.3, 0.5 or 1 mM phlorizin in the mucosal chamber, followed by a 45 mM d‐glucose challenge.

### 2.6 Study 3: oestrous cycle

To determine any effect(s) of oestrous cycle stage on SGLT1‐mediated jejunal glucose transport, the oestrous cycle was tracked in mice via daily vaginal smears (Caligioni, 2009) for at least a week. Mice were humanely killed by cervical dislocation at pro‐oestrus, dioestrus, metoestrus and oestrus stages (*n* = 9–10 per stage) and jejunum segments incubated for 20 min with 0, 0.1, 0.3 or 1 mM phlorizin in DMSO vehicle (1% v/v ) followed by a 45 mM d‐glucose challenge.

### 2.7 Statistical analysis

Data were analysed using the mixed models procedure of SPSS Statistics version 28 (IBM Corp., Armonk, NY, USA), treating data from multiple sections from individual mice as repeated measures. Glucose dose–response data (Study 1) were analysed for effects of region (proximal duodenum, distal duodenum, proximal jejunum, distal jejunum, proximal ileum, distal ileum), glucose concentration and interaction. DMSO response data (Study 2) were analysed for effects of region (proximal duodenum, distal duodenum, proximal jejunum, distal jejunum, proximal ileum, distal ileum), DMSO concentration and interaction. Phlorizin response data (Study 2) were analysed for effects of region (proximal or distal jejunum), phlorizin concentration and interaction. Oestrous cycle data (Study 3) were analysed for effects of cycle stage, region (proximal or distal jejunum), phlorizin concentration and interactions. Negative short‐circuit current responses in some experiments with inhibitors were corrected to a minimum response of 0.001. Data were log‐transformed before analysis to reduce inequality of variance between groups. All data are presented as means ± standard deviation and individual responses, and *P* < 0.05 is accepted as statistically significant.

## 3 RESULTS

### 3.1 Study 1: region‐specific glucose dose response

**FIGURE 1:**
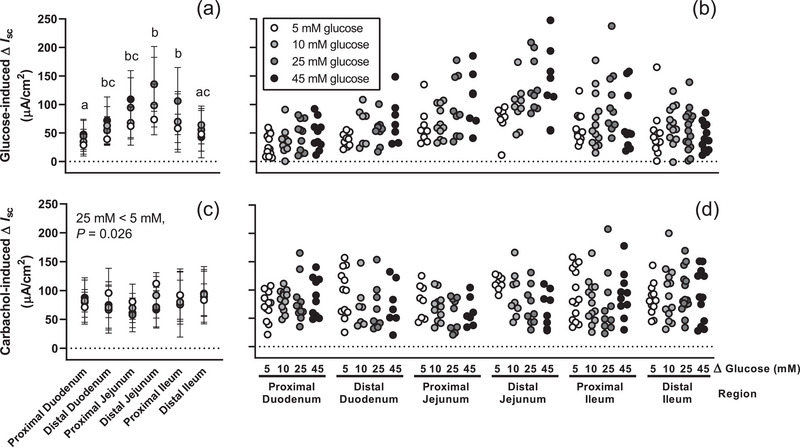
Glucose dose–response. Mean (a) and individual (b) changes in short‐circuit current (∆*I*_sc_) induced by 5, 10, 25 or 45 mM d‐glucose were assessed in each small intestine region of 9‐ to 10‐week‐old C57BL/6 female mice. Peak active glucose transport occurred in the presence of 50 mM d‐glucose in the distal jejunum (a,b). Tissue viability assessed by short‐circuit current responses to 100 µM carbachol did not differ by region, but was lower following challenge with 25 mM than 5 mM glucose (c,d). Responses to glucose or carbachol were assessed by repeated measures mixed model for effects of region and glucose concentration (*n* = 7–15 mice/group). The change in short‐circuit current differed between regions that do not share a common lowercase letter (*P* < 0.05, panel a).

The length of the small intestine in glucose dose–response experiments averaged 31.9 ± 1.5 cm. Short‐circuit current responses to glucose differed between regions (*P* < 0.001) and glucose concentrations (*P* = 0.002), with the greatest responses seen in distal jejunum (Figure 1a,b). Responses to addition of 25 mM (*P* = 0.004) or 45 mM (*P* = 0.010) were greater than those generated by addition of 5 mM glucose (Figure 1a,b). Short‐circuit current responses to carbachol (Figure 1c,d) did not differ between regions (*P* = 0.159), but were affected by preceding glucose dose (*P* = 0.034). Responses to carbachol were lower following a glucose challenge with 25 mM glucose than at 5 mM glucose (*P* = 0.026) but did not differ between any other dose pairs (Figure 1c,d).

### 3.2 Study 2: SGLT1 inhibition

**FIGURE 2:**
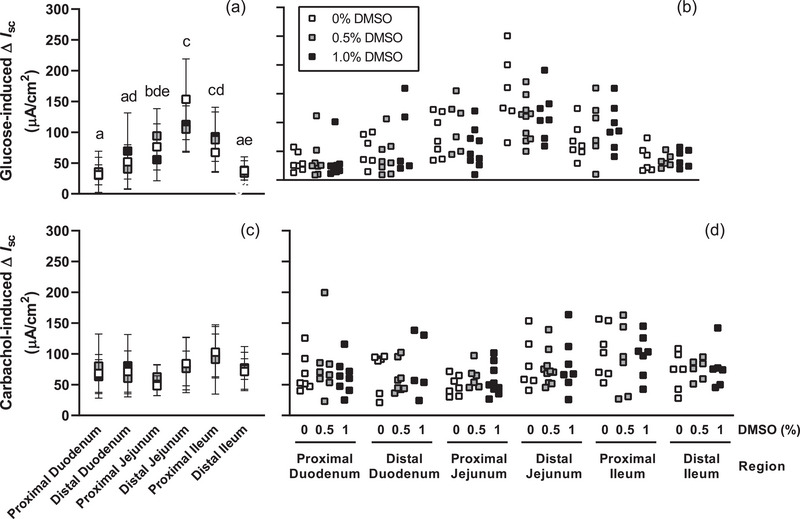
DMSO does not inhibit glucose or carbachol responses. Mean (a) and individual (b) changes in short‐circuit current (∆*I*_sc_) induced by 45 mM d‐glucose in the presence of 0%, 0.5% or 1% DMSO were assessed in each small intestine region of 9‐ to 10‐week‐old C57BL/6 female mice. DMSO did not affect short‐circuit current responses to glucose (a,b) or carbachol (c,d) challenge. Responses to glucose or carbachol were assessed by repeated measures mixed model for effects of region and DMSO concentration (*n* = 5–10 mice/group). The change in short‐circuit current differed between regions that do not share a common lowercase letter (*P* < 0.05, panel a).

The length of the small intestine in experiments to evaluate effects of DMSO averaged 32.3 ± 1.9 cm. Our initial experiments confirmed that short‐circuit current responses to glucose (*P* = 0.788) and carbachol (*P* = 0.966) were not altered by addition of DMSO to the incubation medium (Figure 2). Responses to glucose varied between regions (*P* < 0.001), and were again greater in the distal jejunum (Figure 2a,b). Short‐circuit current responses to carbachol (Figure 2c,d) did not differ between regions (*P* = 0.056). Given the markedly greater glucose transport in jejunum, subsequent experiments were conducted in this region.

**FIGURE 3:**
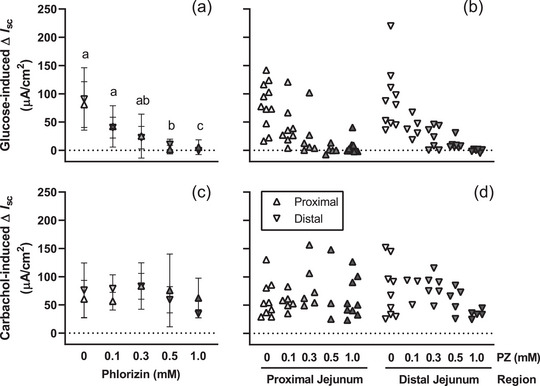
Phlorizin inhibits active glucose uptake. Mean (a) and individual (b) changes in short‐circuit current (∆*I*_sc_) induced by 45 mM d‐glucose in the presence of 0, 0.1, 0.3, 0.5 or 1 mM phlorizin were assessed in proximal and distal jejunum of 9‐ to 10‐week‐old C57BL/6 female mice. Phlorizin inhibited short‐circuit current responses to glucose in a dose‐dependent manner (a,b), but did not affect responses to carbachol (c,d). Responses to glucose or carbachol were assessed by repeated measures mixed model for effects of region and phlorizin concentration (*n* = 5–11 mice/group). The change in short‐circuit current differed between phlorizin concentrations that do not share a common lowercase letter (*P* < 0.05, panel a).

The length of the small intestine in experiments to confirm inhibition of active glucose transport by phlorizin averaged 33.0 ± 1.0 cm. Addition of phlorizin to the incubation medium attenuated short‐circuit current responses to glucose (*P* < 0.001, Figure 3a,b). Short‐circuit current responses to glucose (*P* = 0.086), and effects of phlorizin (*P* = 0.055), were similar in proximal and distal jejunum. Short‐circuit current responses to carbachol (Figure 3c,d) were unaffected by phlorizin (*P* = 0.970) or region (*P* = 0.096).

### 3.3 Study 3: oestrous cycle

**FIGURE 4:**
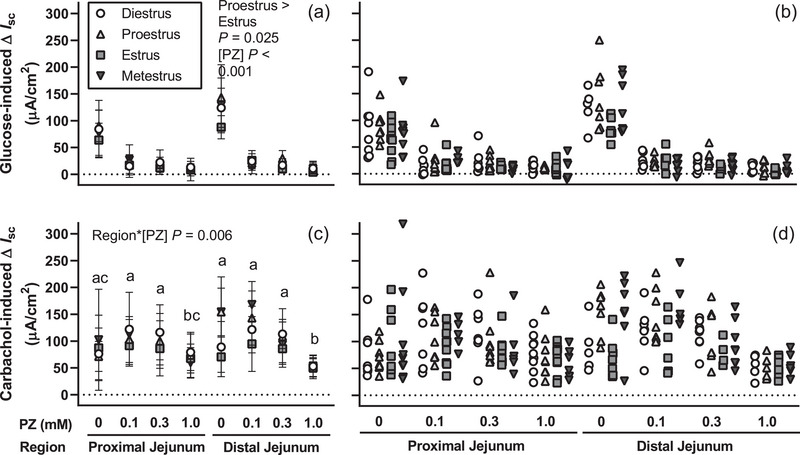
Active glucose uptake varies across the oestrous cycle in mice. Mean (a) and individual (b) changes in short‐circuit current (∆*I*_sc_) induced by 45 mM d‐glucose in the presence of 0, 0.1, 0.3 or 1 mM phlorizin (PZ) were assessed in proximal and distal jejunum of 9‐ to 10‐week‐old C57BL/6 female mice at each oestrous cycle stage. Active glucose transport was lower at oestrus than pro‐oestrus (*P* = 0.025, a,b). Phlorizin inhibited short‐circuit current responses to glucose (*P* < 0.001, a,b). Incubation with 1 mM phlorizin reduced short‐circuit current responses to carbachol in the distal jejunum (c,d). Responses to glucose or carbachol were assessed by repeated measures mixed model for effects of oestrous cycle stage, region and phlorizin concentration (*n* = 5–9 mice/group). Within each region, the change in short‐circuit current differed between phlorizin concentrations that do not share a common lowercase letter (*P* < 0.05, panel c).

The length of the small intestine did not differ between oestrous cycle stages (dioestrus: 31.1 ± 1.4 cm; pro‐oestrus: 31.7 ± 1.8 cm; oestrus: 31.1 ± 1.8 cm; metoestrus: 31.9 ± 1.6 cm; *P* = 0.579). Short‐circuit current responses to glucose changed across the oestrous cycle (*P* = 0.030) and were higher at pro‐oestrus compared to oestrus (*P* = 0.025) but did not differ between other stages (Figure 4a,b). Responses to glucose were similar in the proximal and distal jejunum (*P* = 0.989) and were attenuated by phlorizin (*P* < 0.001) with the highest phlorizin concentration tested (1 mM) reducing short‐circuit current responses to glucose by ∼90% (Figure 4a,b). All concentrations of phlorizin reduced responses to glucose relative to control (each *P* < 0.001).

Short‐circuit current responses to carbachol did not change across the oestrous cycle (*P* = 0.326), while the effect of phlorizin on response to carbachol differed between regions (interaction *P* = 0.006, Figure 4c,d). Phlorizin affected the response to carbachol in proximal and distal jejunum (each *P* < 0.001), with carbachol responses attenuated at 1 mM phlorizin in both regions (Figure 4c).

## 4 DISCUSSION

This study provides the first direct evidence that active glucose transport by the jejunum varies during the ovarian cycle. This adds support to the hypothesis that differences in post‐prandial blood glucose during the human menstrual cycle (Brennan et al., 2009) reflect changes in glucose uptake, at least in part. This validated *ex vivo* method enables future assessment of region‐specific changes in active glucose transport by mouse small intestine induced by physiological states, environmental challenges and pharmacological agents.

We observed peak short‐circuit current responses to a stimulus of 45 mM glucose in mice, consistent with saturation of SGLT1‐faciliated glucose transport at 30–50 mM glucose in rat jejunum (Kellett & Helliwell, 2000). In Ussing chamber studies using human small intestine, active glucose transport was maximal at 35–65 mM luminal glucose (Hardcastle et al., 1994; Kroesen et al., 2002; Larsen et al., 2001; Puthanmadhom Narayanan et al., 2021). Regional luminal glucose concentrations measured in vivo via indwelling catheters ranged from 2 to 48 mM glucose, in rodents, rabbits and dogs fed standard laboratory diets (Ferraris et al., 1990), confirming that the glucose concentrations we used in Ussing experiments were physiological. Regional differences in active glucose transport across the small intestine were consistent between the present study and that of Smith et al. (2018), using similar methodology in the same strain of mice. We observed peak active glucose transport in the distal jejunum, consistent with previous reports in mice (Smith et al., 2018) and rats (Rider et al., 1967). Corresponding to this regional pattern of active glucose transport, SGLT1 gene and protein expression are higher in jejunum than ileum or duodenum in rat (Haase et al., 1990; Takata et al., 1992; Yoshida et al., 1995) and pig (Klinger et al., 2018). In contrast, SGLT1 gene expression was ∼2.5 and ∼4‐fold higher in the duodenum than jejunum and ileum, respectively in a single study in male BALB/c mice (Yoshikawa et al., 2011). A limitation of our study is that we did not collect tissues to assess gene and tissue expression of SGLT1 after Ussing experiments. Nevertheless, our data on region‐specific active glucose transport, together with gene expression patterns in rats and humans, are consistent with the jejunum as the primary site of glucose uptake across mammalian species. Interestingly, we observed proximal–distal differences in active glucose transport within each small intestinal region, suggesting gradients in function, rather than sharp changes at the boundaries between regions.

Adding up to 1% DMSO to KRB buffers did not alter short‐circuit current responses or tissue viability, reaffirming DMSO as a suitable vehicle for testing pharmacological compounds, such as phlorizin, in epithelial tissues such as the small intestine. In our subsequent experiments, 1 mM phlorizin attenuated active glucose transport by 90–97% in mouse jejunum, consistent with the proportion of SGLT1‐mediated active glucose transport reported in human ileum (Sun et al., 2017). There is some evidence that the facilitative glucose transporter 2 (GLUT2) may translocate from a basolateral location to the brush border membrane to facilitate glucose and fructose absorption (Kellett & Brot‐Laroche, 2005; Kellett et al., 2008). However, this has been reported in limited settings – severely insulin resistant non‐diabetic mice fed a high‐fat, low‐carbohydrate diet for 12 months starting from weaning, and in morbidly obese human subjects (Ait‐Omar et al., 2011; Kellett & Brot‐Laroche, 2005; Kellett et al., 2008). Indeed, individuals with inactivating mutations in GLUT2 (Fanconi–Bickel syndrome) do not have defective intestinal glucose uptake (Santer et al., 2003). Our data thus support evidence that GLUT2 does not play a major role in glucose transport under normal circumstances. We did observe that short‐circuit current responses to carbachol were lower in the presence of 1 mM phlorizin than at lower phlorizin concentrations in oestrous cycle experiments, suggesting that 1 mM phlorizin may compromise tissue viability. Addition of 0.3–0.5 mM phlorizin inhibited 73–83% of active glucose transport without loss of carbachol response. We therefore suggest using a maximum concentration of 0.5 mM phlorizin in mouse intestine.

Active glucose transport across the jejunum in our study was lower at oestrus compared to pro‐oestrus, while short‐circuit current responses to carbachol did not differ with cycle stage. This finding supports the hypothesis that the rate of small intestinal glucose transport is higher during the luteal than follicular phase of the mammalian ovarian cycle. Interestingly, we only saw differences in glucose transport between the late follicular (pro‐oestrus) and early luteal (oestrus) stages of the murine oestrous cycle. In cycling women, blood glucose concentrations after ingestion of a liquid test meal were lower during the mid‐follicular phase compared to the luteal phase, around a week after ovulation (Brennan et al., 2009). The mechanisms underlying changes in active glucose transport during the ovarian cycle are unclear, but may include changes in food intake, gastric emptying and circulating hormones, or a combination of factors. The small intestine did not differ in length between cycle stages, concurrent with altered glucose transport. Although individual food intake, body mass and hormone concentrations were not assessed in the present study, future investigation of these across the oestrous cycle may assist in identifying the underlying mechanisms. Further studies are also required to determine whether active glucose transport varies across the ovarian cycle during the dark period, when food intake and SGLT1 expression peak in mice (Iwashina et al., 2011; Zhong et al., 2019). The lower active glucose transport observed at oestrus in the current study corresponds to the timing of nadirs in meal size and duration reported previously in mice (Petersen, 1976), rats (Drewett, 1974) and humans (Lissner et al., 1988). A role for hormonal control is supported by the finding that lower blood glucose concentrations after a test meal corresponded to higher circulating oestrogen during the follicular phase than luteal phase in cycling women (Brennan et al., 2009). In rats, small intestinal glucose uptake was increased following ovariectomy but normalised to that of sham‐operated controls following 2 weeks of 17β‐estradiol and/or progesterone treatment at physiological or pharmacological doses (Singh et al., 1985). Together, these data suggest a high plasticity of glucose transport, potentially driven by cyclic changes in circulating sex steroids, and corresponding to changes in food intake across the oestrous cycle in rats and mice. Whether glucose transport is perturbed in conditions of dysregulated ovarian cycles, such as polycystic ovarian syndrome, or by endocrine‐disrupting chemicals with oestrogenic activity, is yet to be determined.

In conclusion, our study provides the first direct evidence of changes in SGLT1‐mediated glucose transport in the jejunum across the ovarian cycle. We next plan to use this validated method to investigate whether region‐specific active glucose transport changes during sustained physiological states in which food intake and circulating hormones change, such as during pregnancy and lactation.

## AUTHOR CONTRIBUTIONS

Conception or design of the work: T. Sebastian Overduin, Richard L. Young, Amanda J. Page, Kathryn L. Gatford Acquisition, analysis, or interpretation of data for the work: T. Sebastian Overduin, Hannah R.Wardill, Richard L. Young, Amanda J. Page, Kathryn L. Gatford. Drafting of the work or revising it critically for important intellectual content: T. Sebastian Overduin, Hannah R.Wardill, Richard L. Young, Amanda J. Page, Kathryn L. Gatford. All authors have approved the final version of the manuscript, and agree to be accountable for all aspects of the work in ensuring that questions related to the accuracy or integrity of any part of the work are appropriately investigated and resolved. All persons designated as authors qualify for authorship, and all those who qualify for authorship are listed.

## CONFLICT OF INTEREST

The authors have no conflicts of interest to declare.

## Supporting information


Statistical Summary Document

## Data Availability

The data that support the findings of this study are available from the corresponding author upon reasonable request.
